# Mechanisms of Sterile Inflammation

**DOI:** 10.3389/fimmu.2013.00398

**Published:** 2013-11-22

**Authors:** Anna Rubartelli, Michael T. Lotze, Eicke Latz, Angelo Manfredi

**Affiliations:** ^1^Cell Biology, IRCCS AOU San Martino IST, Genova, Italy; ^2^University of Pittsburgh Cancer Institute, Pittsburgh, PA, USA; ^3^University of Massachusetts Medical School, Worcester, MA, USA; ^4^Institute of Innate Immunity, University of Bonn, Bonn, Germany; ^5^Vita Salute San Raffaele University, Milano, Italy; ^6^San Raffaele Scientific Institute, Milano, Italy

**Keywords:** stress, inflammasome activation, HMGB1, IL-1, DAMPs, PRR, acute inflammation, chronic inflammation

Inflammation is a coordinated response of the immune system which is aimed at maintaining or restoring tissue integrity. Sterile inflammation can be triggered by physical, chemical, or metabolic noxious stimuli. The individual stimuli (genomic stress, ER stress, hypoxic stress, nutrient stress, etc.) promote a carefully choreographed set of cell responses to stress. Many types of stress responses exist (e.g., the unfolded protein response, integrated stress response, oxidative stress response, autophagy, etc.), and these can influence each other. Stress responses induce recruitment of inflammatory cells and result in inflammation. When the noxious stimuli persist over and cannot be eliminated or cleared inflammation fails to resolve, resulting in the development of a vicious circle which is part of the pathophysiology of many human diseases, including cancer, autoimmunity, chronic viral infections, chronic graft versus host disease, metabolic syndromes, and several acquired and inborn genetic disorders. Several proximal factors have been identified and proposed to play a role in the individual types of sterile inflammation, including redox responses, the occurrence of damage-associated molecular patterns molecules (DAMPs) and immune stimulatory heat shock proteins, and vascular remodeling. However, the detailed mechanism(s) linking stressful events and the development of inflammation have thus far remained elusive. The identification of the major molecular species in induction, development, and outcome of sterile inflammation, and the illumination of their mechanisms of action are therefore of paramount relevance for the design of effective therapeutic strategies for the treatment of the most common diseases of the Western world. Thus, this Special Topic focuses on articles that can shed new light on the molecular mechanisms of sterile inflammation. This collection of papers, written by experts in this field, addresses the most important current challenges in the topic of sterile inflammation.

A significant focus is placed on the factors that mediate sterile inflammation: DAMPs, released during tissue injury, and cytokines of the IL-1 family. In this family, some members such as IL-1β and IL-18 are true cytokines, in that they undergo active secretion by inflammatory cells, highly regulated at the post-translational level by inflammasomes (1) and regulatory receptors (2). Others, such as IL-1α (3), are molecules that are both DAMPs and cytokines, in that they initiate and perpetuate inflammation either after active secretion or when released by stressed cells undergoing necrosis (4). Interestingly, a similar behavior features the prototypic DAMP high mobility group box 1 (HMGB1) (5). In addition, this series describes the most recent observations on the cells involved in the process of sterile inflammation, not only professional inflammatory cells such as myelomonocytic cells but also innate lymphoid cells (6), granulocytes (7), and glial cells (8). Finally, sterile inflammation as a mechanism of disease is illustrated in important in pathologies such as type 2 diabetes (9) and endometriosis (10), in fungal infection, where DAMPs cooperate with pathogen associated molecular pattern molecules (PAMPs) in switching protective versus pathogenic inflammation (11), and in the regulation of physiologic processes such as parturition (12).
